# Supplementary material for: A Split Luciferase Complementation Assay for the Quantification of β-Arrestin2 Recruitment to Dopamine D2-Like Receptors
Source: Int J Mol Sci. 2020 Aug 24;21(17):6103. doi: 10.3390/ijms21176103 (PMC7503597; doi:10.3390/ijms21176103)
Supplement: Supplementary file 1 [file ijms-21-06103-s001.pdf]

Supplementary Materials

A Split Luciferase Complementation Assay for the Quantification of β-Arrestin2 Recruitment to Dopamine D_2-like Receptors

Lisa Forster *, Lukas Grätz, Denise Mönnich, Günther Bernhardt and Steffen Pockes *

Institute of Pharmacy, University of Regensburg, D-93053 Regensburg, Germany; lisa.forster@ur.de (L.F.); lukas.graetz@ur.de (L.G.); denise.moennich@ur.de (D.M.); guenther.bernhardt@ur.de (G.B.); steffen.pockes@ur.de (S.P.)

* Correspondence: lisa.forster@ur.de, Tel.: +49-941-943-4796; steffen.pockes@ur.de, Tel.: +49-941-943-4825

Contents

1. Radioligand Saturation Binding Data S2

2. β-Arrestin2 Recruitment Data S3

3. References S3

1. Radioligand Saturation Binding Data

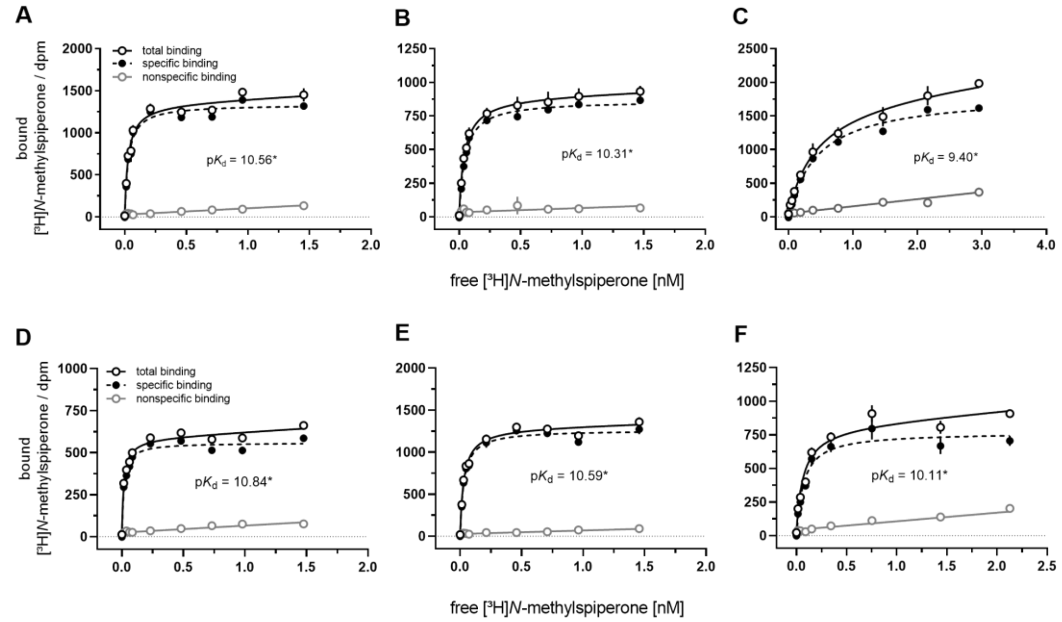

Figure S1. Radioligand saturation binding curves with whole HEK293T ELucN-βarr2 cells expressing the D2longR-ELucC (A), D3R-ELucC (B) or D4.4R-ELucC (C) fusion proteins and homogenates from cells expressing the wild-type D2longR (D), D3R (E) or D4.4R (F). Corresponding dissociation constants are provided in Table 1 in the manuscript. Graphs represent means ± SEM from one representative experiment performed in triplicate of three independent experiments. **pKd* values are given as mean from three independent experiments.

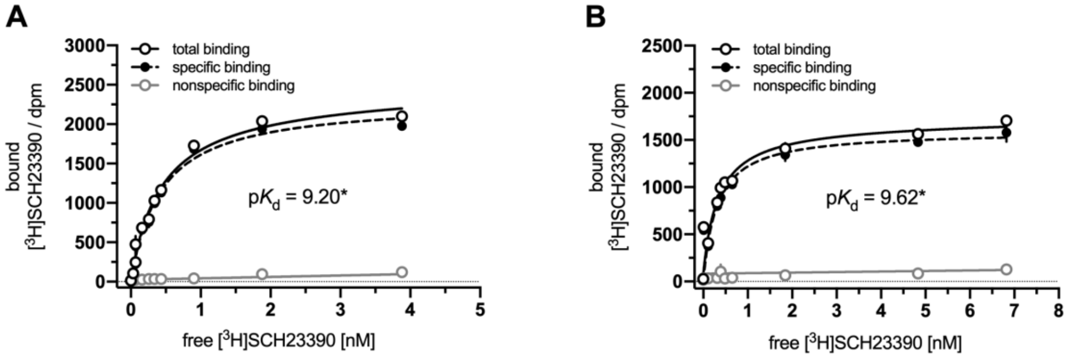

Figure S2. Radioligand saturation binding curves with whole HEK293T ELucN-βarr2 cells expressing the D1R-ELucC (A) fusion proteins and homogenates from cells expressing the wild-type D1R (B). Corresponding dissociation constants are provided in Table S1 in the Supplementary Materials. Graphs represent means ± SEM from one representative experiment performed in triplicate of three independent experiments. **pKd* values are given as mean from three independent experiments.

Table S1. Dissociation constants (pK_d values) of [^{3}H]SCH23390 determined in radioligand saturation binding experiments at receptors fused to the C-terminal fragment of the Emerald luciferase using whole cells and at wild-type receptors using homogenates. Data represent means ± SEM determined in three independent experiments, each performed in triplicate.

	D_1R	
	**ELucC Fusion Protein**	**wt**
pK_d	9.20 ± 0.09	9.62 ± 0.07

2. β-. Arrestin2 Recruitment Data

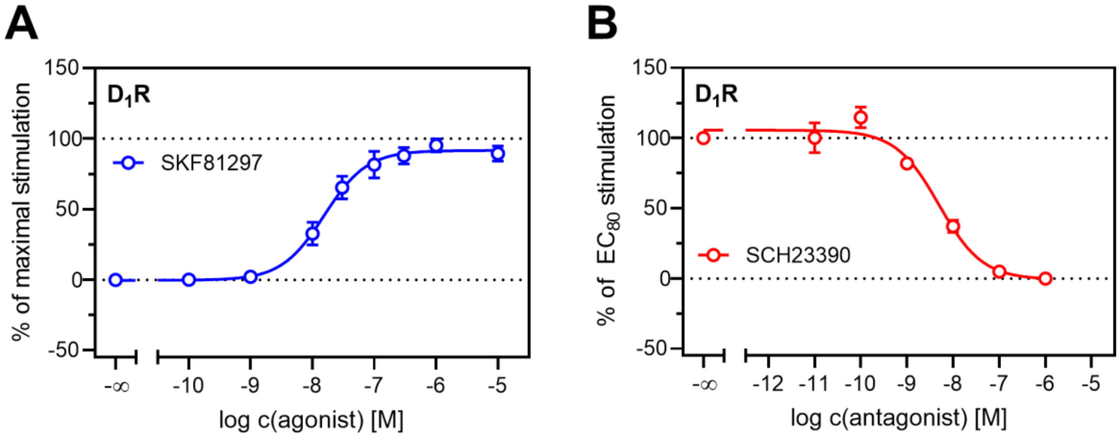

Figure S3. Characterization of the standard agonist SKF81297 and standard antagonist SCH23390 in the β-arrestin2 recruitment assay at the D_1R. Data of the agonist were normalized to the maximal stimulation (100%) and a solvent control (0%). Antagonist data were normalized to the signal elicited by SKF81297 at a concentration corresponding to the EC_{80} (100%) and a solvent control (0%). Obtained pEC_{50} and pK_b values are presented in Table S2. Data represent means ± SEM from at least three independent experiments, each performed in triplicate.

Table S2. pEC_{50}, E_{max} and pK_b values of SKF81297 and SCH23390 analysed in the newly developed β-arrestin2 recruitment assay at the D_1R. For comparison, pK_i values from previously published data are included. Data represent means ± SEM from N independent experiments, each performed in triplicate.

		β-Arrestin2 Recruitment			Ref.
**Receptor**	**cpd**	**pEC_{50}**	**pK_b**	**N**	**pK_i**
**D_1R**	SKF81297	7.75 ± 0.15		3	7.47[1]
	SCH23390		8.84 ± 0.07	3	9.33[2]

3. References

1. Andersen, P. H.; Jansen, J. A. Dopamine Receptor Agonists: Selectivity and Dopamine D1 Receptor Efficacy. *Eur. J. Pharmacol. Mol. Pharmacol.* **1990**, *188* (6), 335–347.
2. Sunahara, R. K.; Guan, H.-C.; O'Dowd, B. F.; Laurier, L. G.; Ng, G.; George, S. R.; Torchia, J. Cloning of the Gene for a Human Dopamine D5 Receptor with Higher Affinity for Dopamine than D1. **1991**, *350*, 614-619.
